# The lived experiences of men who have sex with men when accessing HIV care services in Zimbabwe

**DOI:** 10.4102/hsag.v26i0.1462

**Published:** 2021-04-22

**Authors:** Idah Moyo, Margaret Macherera, Azwihangwisi H. Mavhandu-Mudzusi

**Affiliations:** 1 Office of Graduate Studies and Research, University of South Africa, Pretoria, South Africa; 2 Department of HIV Services, Population Services International, Harare, Zimbabwe; 3 Department of Environmental Science and Health, Faculty of Applied Science, National University of Science and Technology, Bulawayo, Zimbabwe

**Keywords:** HIV care services, experiences, HIV positive, MSM, enabling environment, gay, transgender woman

## Abstract

**Background:**

Key populations such as men who have sex with men are disproportionately affected by human immunodeficiency virus (HIV), yet they are underserved. This vulnerable group also faces stigma and discrimination when utilising the healthcare services. However, to achieve the HIV epidemic control, it is important for them to have access to HIV care services.

**Aim:**

The aim of this study was to explore and describe the experiences of men who have sex with men (MSM) as they accessed HIV care services in healthcare settings in Bulawayo, Zimbabwe.

**Setting:**

The study setting was healthcare facilities (state and private owned) in Bulawayo, Zimbabwe, that offer HIV care services.

**Methods:**

The study used a descriptive phenomenological design targeting self-identified MSM living with HIV. Data were gathered by using in-depth individual interviews that were audio recorded. Data saturation determined sample size. Data were transcribed verbatim and later analysed thematically.

**Results:**

The study revealed that counselling given to MSM was generalised and not individualised. Some clients faced stigma and discrimination after disclosure. Peer and family support were important in the journey to access HIV care services.

**Conclusion:**

An enabling environment was not provided for MSM clients to access HIV care services in the majority of health facilities. This calls for sensitisation and competency clinical training of service providers so that the diverse needs of MSM are met. Peer and family support for MSM needs to be strengthened.

## Introduction

Key populations are disproportionately affected by human immunodeficiency virus (HIV) but under-represented in HIV testing and treatment programmes (World Health Organization [WHO] 2017:15). In spite of the fact that key populations constitute a small proportion of the general population globally, statistics indicate that in 2018, more than half (54%) of the new infections were from key populations and their partners (UNAIDS 2019a). To achieve HIV epidemic control, it is critical that key populations have access to HIV care services, which include testing, treatment and retention in care and maintaining undetectable viral load. In addition, according to UNAIDS (2019a:9), available data indicated that in 2018 the risk of acquiring HIV by men who have sex with other men (MSM) was 22 times higher than amongst all adult men, and 17% of new infections were from this group globally. Despite this, key population groups such as MSM are underserved. The concealed nature of many key populations, stigma and discrimination and criminalisation of their behaviour make it difficult to track various steps along the HIV care continuum (UNAIDS 2019b:6–7).

UNAIDS data show that, in eastern and southern Africa, there has been a significant decline in the number of new HIV infections and AIDS-related deaths. However, in 2018, 25% of the new infections in these regions were from key populations and their partners. According to UNAIDS, it is estimated that 37.9 million people were living with HIV, and there were 1.7 million new infections globally at the end of 2018. The UNAIDS 2019 Data Report highlighted the fact that sub-Saharan Africa is still at the epicentre of the global HIV epidemic, with 61% of all HIV new infections occurring in this region in 2018. The top five countries being Swaziland (27.3%), South Africa (20.4%), Botswana (20.3%), Zimbabwe (12.7%) and Malawi (9.2%) (UNAIDS 2019a:35–73).

Available data on HIV prevalence among the key populations are scanty. According to UNAIDS (2019a:35–73), the following countries had these prevalences in 2018, for gay men and other MSM: Lesotho (32.9%), Zimbabwe (31%), South Africa (18.1%), Swaziland (12.6%) and Malawi (7%). Across sub-Saharan Africa the HIV prevalence was estimated to be 5% in the general population and 17.9% amongst the MSM (Beyrer et al. 2012:367–377). In the case of West Africa where few studies have been published, a similar picture of higher HIV prevalence among MSM emerges as well, with HIV prevalence estimates of 23% amongst MSM in Nigeria (Eluwa et al. 2019:4).

According to the Zimbabwe Ministry of Health and Child Care and National AIDS Council situation (2018:1), Zimbabwe had approximately 1.3 million people living with HIV in 2018. According to the Zimbabwe Ministry of Health and Child Care (2019:43), the three Matabeleland provinces (amongst which Bulawayo falls) have the highest HIV prevalence in Zimbabwe, disaggregated as follows: Matabeleland North (19.5%), Matabeleland South (21.7%) and Bulawayo Metropolitan (17.9%). Zimbabwe Ministry of Health and Child Care (2017:31) also state that information on HIV prevalence amongst key populations in Zimbabwe is not known. In addition, there is reduced access to HIV service and incomplete documentation on service delivery models for key population groups.

Despite the effective implementation and significant progress made in the prevention, treatment and care programme in Zimbabwe, the key population groups have not been properly embraced. According to UNAIDS (2019a:23), in sub-Saharan Africa epidemiological data on key populations is either scanty or non-existent. Of the two countries that had data in 2018, the HIV prevalence amongst MSM was around 30%. Yet, effective programming targeting this group depends on evidence-based research.

In line with the 95-95-95 UNAIDS strategy and commitments that aim at accelerating HIV response and a reduction in new HIV infections, it is critical that key population groups have access to HIV care services. On the other hand, WHO (2016:4) posits that it is difficult to estimate the full extent of key populations’ access to services because of lack of data and challenges in data disaggregation. Therefore, evidence generated through this study could support policy and programme redesign to ensure accessible and acceptable service delivery for the MSM community. Curbing the HIV epidemic will not be possible without reaching key populations such as MSM (Gupta & Granich 2017:125–132; WHO 2016:4–5). The researchers therefore found it essential to explore the lived experiences of MSM who accessed HIV care services in antiretroviral therapy (ART) sites in Bulawayo. In Zimbabwe, like in most Southern African countries, membership to key population groups carries with it a stigma, a taboo and in some instances, criminalisation. Related to this is the perception that this group is insignificant. Within this given context, it was of interest to understand the lived experiences of this vulnerable group as it accessed HIV care services in Bulawayo.

## Research methods and design

### Study design

The study used descriptive phenomenological design – a type of phenomenology developed by Husserl, whose philosophy emphasised descriptions of human experience (Rees 2011:43–44). On a related note, Englander (2012:25) stated that in phenomenological research the aim is to encounter the phenomenon via the person’s description. In this particular instance, the researchers were interested in understanding the meaning of the experiences of HIV-positive MSM as they access HIV care services.

### Study setting

The study setting was healthcare facilities in Bulawayo, Zimbabwe that offer HIV care services (HIV testing, ART initiation, adherence counselling and viral load monitoring). The city has a number of facilities that are either state or privately owned and provide HIV care services for key populations. The service providers are nurses and primary care counsellors, and the medical practitioners only come in to review complicated cases.

### Study population

The study population consisted of HIV-positive MSM and were accessing HIV care services in healthcare facilities in Bulawayo. The inclusion criteria for participating in the study were as follows: being an MSM living with HIV, self-identified as MSM (i.e. men who have sex with other men), aged from 18 and above, receiving HIV care services in healthcare facilities in Bulawayo, Zimbabwe, and have been on ART for a minimum period of 2 years or less.

### Study sample

To gain access to this hard-to-reach population (MSM), one of the organisations that provides services for key populations and runs a support group for this community was requested to recruit participants. The researchers were able to gain access to the initial participants. Snowball sampling was then used to identify potential interviewees through the networks of those who had already been interviewed. The participants were asked to consider if there was anyone else (MSM) in their networks who they thought would be interested in participating in the study and inform them about the research. Judgemental purposive sampling was used to recruit participants for this study (Borbasi & Jackson 2012:135). The participants met the inclusion and exclusion criteria. If they agreed, their contact details were then passed on to the researchers for recruitment purposes. Potential participants were told that the study was being conducted to learn more about MSM’s experiences and challenges in accessing HIV care services. All participants were assured that their participation and responses would be anonymised, and their personal details would not be shared with others. It was not possible to recruit the study participants from the health facilities where they accessed HIV care services, because of the socio-political environment. The sample size of 15 participants was determined by data saturation (Mason 2010:2; Nakkeeran & Zodpey 2012:8).

### Data collection

Data were collected from 15 December 2018 to 15 March 2019. Face-to-face interviews conducted by the researchers were unstructured, in-depth and open ended. All study participants were interviewed by two researchers, experienced in qualitative research, who had the competence and experience of working with MSM. To ensure privacy and confidentiality, the clients were interviewed in the local language (Ndebele) in a selected private room at the centre. To facilitate authenticity and a clear audit trail, the interviews were audio recorded and later transcribed. Apart from the central research question, probing questions such as the following were also asked to elaborate on or clarify statements: From your point of view, what are the factors that facilitated or impeded your access to these HIV-related healthcare services? Then what happened? What were your feelings?

Researchers also kept field notes. A reflexive diary was utilised to ensure that all questions in subsequent interviews were open ended. The duration of each interview was about 30 to 60 min. A pilot study that involved four MSM clients was conducted in one of the centres that provide HIV services for key populations. This process helped to refine the research instrument and to further rephrase some of the follow-up questions. The researchers greeted each participant, explained the purpose of the study as well as interview and obtained a written informed consent before proceeding with the data collection and audio-recording of the sessions. Research participants were encouraged to keep all information shared during the interview session confidential.

### Measures to ensure trustworthiness

Measures were taken to ensure trustworthiness and rigour (Moule & Goodman 2014:188). A reflective diary was kept ensuring neutrality and objectivity. To enhance credibility, prolonged engagement, data triangulation, peer evaluation and a co-coder were used. The third researcher, who was not involved in data collection acted as an independent co-coder. Records were kept locked in a cupboard, thus ensuring that an audit trail was left to be followed by any researcher. As part of the evaluation, peer debriefing was performed and irregularities identified were addressed. By coding and recoding many times, comparing the themes and categories with a co-coder, researchers ensured dependability. To enhance authenticity, verbatim extracts from the interviews were utilised.

### Data analysis

All researchers, working independently transcribed the audio recordings verbatim and translated them from Ndebele to English. The two researchers who collected data, and the third one who acted as an independent coder conducted open coding of each transcript. The data were analysed by using interpretive phenomenological analysis (IPA). The following steps as outlined by Langridge (2007:13) were followed: (1) reading and re-reading the transcript, (2) note taking and developing emergent themes, (3) clustering the emergent themes, (4) crafting a master table of themes composed of superordinate themes, subthemes and extracts from the interviews, (5) examining and comparing the similarities between the master tables of the themes and (6) compiling a single master list composed of a superordinate theme, themes and sub-themes. The research team worked collaboratively, had a consensus discussion and agreed on the final themes.

### Ethical considerations

The study protocol, informed consent documents and interview topic guides were reviewed and approved by the Medical research Council of Zimbabwe. Ethical clearance was provided by the Medical research Council of Zimbabwe (Ethics Clearance Number MRCZ/A/2338) and by the Ministry of Health and Child Welfare. Authority to gain access to the study participants was obtained from the Sexual Rights Centre. Anonymity and confidentiality were observed as the names of neither the participants nor institutions were used. Only the researchers reviewed and transcribed the data.

All participants completed and provided written informed consent before beginning to participate in the study. As homosexuality is illegal in Zimbabwe, participants were asked to use pseudonyms during the consenting process to protect their identity. Code numbers were assigned to every participant for reference during discussion. Researchers clearly explained that participation was purely voluntary and the participants were free to decline or discontinue the participation at any time if they wished to do so.

## Results

### Biographical information

The sample consisted of 15 participants aged between 25 and 45 years and self-identified as MSM. The study participants had accessed services from six facilities, five of which were public sector facilities and one was privately owned. None of the study participants were married nor formally employed. Table 1 shows the biographic data of study participants.

**TABLE 1:** Interviewee category.

Interviewee code	Age range (years)	Gender	Level of education	Type of facility	Duration on ART (months)
M001	36‒40	Cisgender man	‘O Level’	State-owned facility	18
M002	41‒45	Cisgender man	Primary	State-owned facility	20
M003	36‒40	Cisgender man	‘O Level’	Private clinic	12
M004	36‒40	Transgender woman	Tertiary	State-owned facility	13
M005	36‒40	Cisgender man	‘O Level’	Private clinic	18
M006	31‒35	Cisgender man	‘O Level’	Private clinic	15
M007	25‒30	Cisgender man	Tertiary	Private clinic	11
M008	25‒30	Cisgender man	‘O Level’	State-owned facility	14
M009	25‒30	Cisgender man	‘O Level’	State-owned facility	12
M0010	31‒35	Cisgender man	Tertiary	State-owned facility	18
M0011	31‒35	Cisgender man	‘A Level’	State-owned facility	17
M0012	36‒40	Transgender woman	‘O Level’	Private clinic	18
M0013	25‒30	Cisgender man	‘A Level’	State-owned facility	19
M0014	31‒35	Cisgender man	‘O Level’	State-owned facility	24
M0015	31‒35	Cisgender man	‘O Level’	Private clinic	21

ART, antiretroviral therapy.

From the exploration and description of participants’ experiences as they accessed HIV care services, four themes and related sub-themes emerged as outlined in Table 2.

**TABLE 2:** Summary of themes and sub-themes.

Themes	Sub-themes
Counselling content	Generalised counselling
	Client-centred counselling
Disclosure/non-disclosure about sexual orientation	Safe and conducive environment for disclosure
	None-conducive environment for disclosure
Stigma and discrimination	Stigmatising attitudes of services providers
	Unfriendly and non-inclusive health settings
Support system	Peer support
	Family support
	Lack of support system

#### Theme 1: Counselling content

Two sub-themes emerged: generalised and client-centred counselling.

### Generalised counselling

The findings indicate that counselling provided in public health sector facilities was generalised, not individualised, and focussed mainly on HIV care-related issues. The counselling was often framed in terms that assumed clients were in monogamous heterosexual relationships as illustrated by the following extracts from the interviews:

‘I had a stomach ache and went to the clinic. I was given treatment for 7 days and was to be reviewed a week later. I was seen by this nurse who kept on asking me where my wife was. Unfortunately, when I came back after 7 days, I had developed herpes zoster. I met the same nurse; she was criticising me and asking me to bring my wife. I felt insulted by this nurse who was assuming that every man should be married.’ (Participant number M004, male sex worker, date of response 18 January 2019)

‘I went through counselling and was started on ART. Counselling focussed on ART, adherence, keeping review dates, sexual health between a man and a woman. Some of this was not applicable to me as I had lied. I just pretended to go along.’ (Participant number M002, male sex worker, date of response 05 February 2019)

### Client-centred counselling

Although the majority of clients had negative experiences about counselling, there were still some cases of positive experiences from a private clinic as demonstrated here:

‘The nurse was very accommodative. I was tested and initiated on ART on the same day. I was treated with dignity. I am able to share information about my private life and the counsellor gives me good and relevant advice. The nurse at the centre encouraged me to bring my partner for testing, which I did. My partner also tested HIV positive and is taking ART at this centre.’ (Participant number M003, self-employed, date of response 19 December 2018)

‘I was free to disclose my sexual orientation because the environment was conducive. I got counselled on how I should take my pills and to practise safer sex.’ (Participant M005, artisan, date of response 04 February 2019)

#### Theme 2: Disclosure about sexual orientation

Two sub-themes emerged describing the experiences on conduciveness or non-conduciveness of the environment for disclosure.

### Safe and conducive environment for disclosure

A convenient and enabling environment at a health facility was found to be a contributor for a client to talk about their sexual orientation as reflected by the following extract:

‘Yes, I shared my sexual orientation with the nurse. However, earlier at the local clinic I had not disclosed. I found the environment conducive for me to disclose.’ (Participant M0012, male sex worker, date of response 19 December 2018)

The findings also underscore the fact that trust and positive rapport have to be cultivated before they can disclose. The delay on disclosure is illustrated by the following extract:

‘I disclosed my sexual orientation, 2 months after starting ART. After the continued interaction with the ART nurse I developed trust and I was able to talk about my sexual orientation and she was supportive.’ (Participant M007, 25–30 years, 11 months on ART, private clinic)

‘I disclosed my sexual orientation on my second visit at the ART facility. The nurse made an indication of the importance of talking about the subject for better care. I was treated just like any other client and did not feel discriminated.’ (Participant number M006, unemployed, date of response 13 February 2019)

### None-conducive environment for disclosure

On the other hand, where the environment was not conducive clients opted not to disclose their sexual orientation as shown here:

‘There was no way I would have disclosed my sexual orientation because I was never asked.’ (Participant M008, accounts clerk, date of response 08 February 2019)

‘I am a gay man, but I did not disclose that at the clinic because of the negative experiences from other clients.’ (Participant M009, footballer, date of response 20 January 2019)

Although a few clients were open about their sexual orientation, the majority lived a dual life of pretence as shown here:

‘They do not know. One time I felt challenged, when I was asked about marital status. I told the nurses that I was single and had a child, telling them lies, because it seems health workers have specific laid questions that they should ask clients. It was not easy to disclose that I am gay. The clinic environment was not comfortable for me to disclose; I had this fear about disclosing this.’ (Participant M0010, photographer, date of response 18 December 2018)

‘I take my drugs well and my health is great, care for me is alright. However, I have lived a life of pretence as I felt I would benefit by not disclosing.’ (Participant M0011, self employed, date of response 08 January 2019)

#### Theme 3: Stigma and discrimination

From the theme on the stigma and discrimination experiences, two sub-themes were elicited, one associated with attitudes of healthcare workers and another illustrating the unfriendly and non-inclusive settings.

### Stigmatising attitudes of services providers

The majority of the research participants never disclosed their sexual orientation. On the other hand, the few who had disclosed had a different experience and felt that they were discriminated by the service providers as displayed by the following extract:

‘After disclosing my sexual orientation to the nurse at the clinic (1 year later), the nurse called other nurses and started to laugh. They were passing funny comments such as: *is this something that exists in this country (being gay)?* They were blaming me for being gay and being HIV positive. They started asking me funny and embarrassing questions.’ (Participant M003, self-employed, date of response 19 December 2018)

Another client who visited a clinic for the treatment of an anal sexually transmitted infection experienced some discrimination as reflected in the following extract:

‘The nurse asked me how I had got this kind of STI (anal). The nurse further called another nurse to come and witness, she literally stated “come and see this strange thing”. The other nurse also commented as “why do you get involved in such behaviours?” After all this drama, I did not get assistance.’ (Participant M0013, unemployed, date of response 01 March 2019)

### Unfriendly and non-inclusive healthcare settings

It is evident that clients belonging to the key population groups experienced many forms of discrimination. One client expressed how painful his journey was as he accessed HIV care services:

‘My journey has been ok; however, initially it was terrible. There was this particular nurse, who would ask me why I am not in a relationship with a female and why I was gay. She would criticise me about being gay. As a result, when my review date was approaching, I would have this fear when I think of going to the clinic. I developed some form of depression …’ (Participant M0014, unemployed, date of response 15 March 2019)

#### Theme 4: Support system

The following sub-themes emerged as part of the support system: peer and family support and lack of both.

### Peer support

The issue of a support system is a critical component for clients receiving HIV care. The majority of MSM reported having received support from friends or peers from the same community, an institution such as Sexual Rights Centre (SRC). The given extract reveals that:

‘The SRC also provides support, we have a support group called ‘Our voice Project’, we motivate each other and share experiences with other gay friends. In this support group we also discuss adherence-related issues, set alarm to remind each other to take ART. I am currently doing well and virally unsuppressed. Over and above the peer support, the SRC also organises a network of medical professionals to attend to our ailments.’ (Participant M0015, self-employed, date of response 15 March 2019)

### Family support

A significant proportion mentioned having obtained support from family members as shown here:

‘My mother is my pillar of strength. My mother has always been there to support me when I am sick.’ (Participant M0012, unemployed, date of response 20 December 2018)

‘My uncle is my treatment assistant, but he doesn’t know that I am gay. He may be suspecting.’ (Participant M009, footballer, date of response 20 January 2019)

### Lack of support system

Half of the research participants stated that gay life was difficult and felt that service providers at ART facilities did not understand them and this interfered with treatment. One example was expressed thus:

‘… but, there were moments I stopped going to the hospital and tempted to default because of my own stressful gay life and the feeling that nurses and the people in general do not understand me.’ (Participant 001, unemployed, date of response 04 February 2019)

## Discussion

The key study findings were that public health facilities were marked by heteronormative counselling, stigma and discrimination and an environment not conducive for the disclosure of sexual orientation. However, in the privately owned clinics the situation was different.

The findings of this study showed that the current quality of counselling in HIV care settings needs to be improved and be more inclusive to meet the dynamic health needs of MSM. These findings demonstrate the need for deliberate efforts to better align health service provision with the concept of differentiated care, particularly in the public sector. A related study in China confirmed that it was imperative to improve the quality and content of counselling services to enhance MSM linkage to HIV care services. In addition, this study highlighted the need for specific efforts in the form of sensitisation and training to address judgemental attitudes amongst service providers (Li et al. 2017:5–6). Key to this would be to conduct clinical competency training that will enable providers to obtain relevant clinical histories. This study also showed that care and/or counselling was generalised and provided in a standardised manner, with particular reference to state-owned facilities. This concurs with the findings of studies in the region where services were found inappropriate and insensitive to the needs of key populations and where there was inadequate access to tailored information and/or services for prevention, treatment and care (Ayala & Santos 2016:1; Duby et al. 2018:3–8; Graham et al. 2018:S97–S105). Of note are the recommendations of WHO (2017:15–19) and Zimbabwe Ministry of Health and Child Care (2017:68) that spell out the need for differentiated HIV care amongst different populations.

Stigma and discrimination discourage clients from accessing HIV care services. Studies on stigma and discrimination and health-seeking behaviour show that people living with HIV who perceive high levels of HIV-related stigma are 2.4 times more likely to delay enrolment in care until they are very ill (Altman et al. 2012:439–445; UNAIDS 2017:2–4). In this study, only a few clients disclosed their sexual orientation and suffered stigma and discrimination. Another client, who by his physiological markers (anal STI) was evidently gay, was also discriminated against.

Related studies have found that the majority of patients were more likely to disclose to healthcare providers with whom they have had a long positive relationship (Furlotte et al. 2016:439–440; Rose, Ussher & Perz 2017:1). The majority lived a dual life of pretence and felt that the environment was not conducive to disclose. These findings concur with the findings of studies in Kenya, Malawi, Namibia and Botswana by Fay et al. (2011:1) and Graham et al. (2018:4–7) that showed that most men from the MSM community felt uncomfortable discussing sexual risk behaviour with providers and preferred that interactions focus on adherence and other aspects of healthcare instead.

It was evident that clients who had disclosed their sexual orientation experienced many forms of discrimination, for example verbal annoying comments from service providers. In addition, the language used by providers in public sector facilities was not always inclusive, hence perceived to be discriminatory by participants. Similar or related findings were established in a study elsewhere in Zimbabwe where stigma and discrimination in healthcare facilities were a major barrier for clients to access HIV care services (Hunt et al. 2017:6–7).

Studies have shown that if the environment is not enabling and key populations are limited in their ability to disclose their sexual orientation, it becomes difficult to appropriately serve this population despite the high HIV incidence (Charurat et al. 2015:8). The reasons for non-disclosure of sexual orientation in this study were the perceived outcomes of discrimination such as receiving poor or unequal care as experienced by other clients in the same community. Similar findings were established in a study in Uganda (Wanyenze et al. 2016:8–15).

In terms of the support system, the findings of this study showed that for MSM clients who were receiving HIV care services, the majority received support from their peers as individuals or in a support group through a non-governmental organisation that supports key populations. These finding are similar to studies elsewhere where peer support played a significant role; for example, the strategy demonstrated to effectively support adherence to ART and sustain retention in care over time and promote good clinical outcomes (Adebajo et al. 2015:555–560; Genberg et al. 2016:7–9; Graham et al. 2018:6–7). The few clients who obtained support from family members had not disclosed their sexual orientation; hence, the focus of the support was on adherence to ART.

## Limitations and strengths

Because the study was confined to facilities in Bulawayo, using snowballing technique, there is a possibility that the findings relate to only people in the same network. This may mean that the MSM who do not belong to that circle of friends could have been left out. Because homosexuality is illegal in Zimbabwe, some participants might have been reluctant to participate. However, the researchers tried their best to emphasise the issues of anonymity and confidentiality to ensure the safety of the participants. The findings will be useful, as it is the first study to the researchers’ knowledge to present a unique qualitative study that is meant to enhance access to HIV care services for clients in the MSM community in Zimbabwe and related contexts.

## Recommendations

To reduce homoprejudice amongst healthcare workers in Zimbabwe, there is need for some training that sensitises them about the specific needs of key populations and fosters an environment that does not assume heterosexuality in all patients. In addition, service providers who are already providing HIV care services to key populations can also share best practices in an effort to improve service quality for the MSM community. This should be coupled with clinical competency training that addresses communication, appropriate health history taking and inclusive but individualised counselling. Of importance would be supporting disclosure of sexual orientation and the need for job aids and training to support disclosure. To enhance linkage to and retention in care for MSM clients, there is need for demand creation for HIV prevention, treatment and care services for this group. This talks about the need for further strengthening of peer-led interventions such as the use of peer navigators to facilitate linkage to care and use of drop-in centres for psychosocial support. In addition, there is a need to create an enabling environment coupled with trust of service providers for clients to be able to freely talk about their sexual orientation. The need for providing a client-centred differentiated service delivery is critical as advocated for by UNAIDS (2019b:115–120), with an emphasis of placing communities at the centre of care, breaking barriers and increasing access to HIV services.

## Conclusion

This is one of the relatively few studies to describe access to and experiences of MSM in accessing HIV care services in Africa. The study findings established that an enabling environment was not always provided to this vulnerable group, service provision was generalised and clients were stigmatised. The findings inform recommendations to provide a safe and confidential environment that facilitates disclosure and ensures access to effective appropriate differentiated treatment and care. Awareness can be raised through educational programmes to dispel myths associated with key populations, reduce stigma and enhance sensitive clinical history-taking skills.
